# Plasma levels of a methadone constant rate infusion and their corresponding effects on thermal and mechanical nociceptive thresholds in dogs

**DOI:** 10.1186/s12917-020-02735-3

**Published:** 2021-01-18

**Authors:** T. Amon, S. B. R. Kästner, M. Kietzmann, J. Tünsmeyer

**Affiliations:** 1 Small Animal Clinic, University of Veterinary Medicine Hannover, Foundation, Bünteweg 9, 30559 Hannover, Germany; 2 Department of Pharmacology, Toxicology and Pharmacy, University of Veterinary Medicine Hannover, Foundation, Bünteweg 17, 30559 Hannover, Germany

**Keywords:** Methadone, CRI, Pharmacokinetic, Threshold, Dog

## Abstract

**Background:**

The present study aimed to collect pharmacokinetic data of a methadone continuous rate infusion (CRI) and to investigate its effect on mechanical and thermal nociceptive thresholds. Seven, 47 to 54 months old beagle dogs, weighing 9.8 to 21.2 kg, were used in this experimental, randomized, blinded, placebo-controlled crossover study. Each dog was treated twice with either a methadone bolus of 0.2 mg kg^− 1^ followed by a 0.1 mg kg^− 1^ h^− 1^ methadone CRI (group M) or an equivalent volume of isotonic saline solution (group P) for 72 h. Mechanical and thermal thresholds, as well as vital parameters and sedation were measured during CRI and for further 24 h. Blood samples for methadone plasma concentrations were collected during this 96 h period.

**Results:**

Percentage thermal excursion (%TE) increased significantly from baseline (BL) until 3 h after discontinuation of CRI in M. Within P and between treatment groups differences were not significant. Mechanical threshold (MT) increased in M until 2 h after CRI discontinuation. Bradycardia and hypothermia occurred in M during drug administration and dogs were mildly sedated for the first 47 h. Decreased food intake and regurgitation were observed in M in five and four dogs, respectively. For methadone a volume of distribution of 10.26 l kg^− 1^ and a terminal half-life of 2.4 h were detected and a clearance of 51.44 ml kg^− 1^ min^− 1^ was calculated. Effective methadone plasma concentrations for thermal and mechanical antinociception were above 17 ng ml^− 1^.

**Conclusion:**

A methadone CRI of 0.1 mg kg^− 1^ h^− 1^ for 3 days after a loading dose results in steady anti-nociceptive effects in an acute pain model in healthy dogs. Main side effects were related to gastrointestinal tract, hypothermia, bradycardia and sedation.

## Background

Methadone, a synthetic opioid analgesic with morphine-like properties, is frequently used for the treatment of severe acute pain in small animals [1–3]. Its antinociceptive effect is mainly attributed to its full agonism at μ-opioid receptors. In addition, it is a non-competitive N-methyl-D-aspartate (NMDA) receptor antagonist [4] and a serotonin and norepinephrine reuptake inhibitor [5], as well as a α3β4 nicotinic receptor inhibitor [6]. Due to these different analgesic, anti-hyperalgesic or pain modulatory effects, methadone is of gaining interest for complex pain therapy [7, 8]. As it has an effect at multiple sites in the pain pathway, “multimodal” pain management could be achieved by one single drug. Furthermore, as the NMDA-receptor plays an important role in the development of opioid induced hyperalgesia, methadone’s antagonistic action at these receptors might be advantageous compared to opioids like morphine and fentanyl, commonly used in small animal analgesia [9]. However, typical dose dependent adverse effects of μ-opioids have been described after IM or IV bolus injection of methadone in dogs. Methadone increases centrally mediated vagal tone [10] and can thereby reduce heart rate [11–13]. Other dose dependent adverse effects in dogs include behavioural changes like sedation [12–14] or dysphoria, such as vocalization, head movements or restlessness [11, 12, 15]. We hypothesized that dose dependent adverse effects could be reduced by administration via constant rate infusion (CRI), because thereby stable plasma levels can be achieved. In general, high peak plasma levels, as observed initially after bolus administrations, are avoided by CRIs, as well as plasma level drops below the therapeutic range. Furthermore, CRIs can better be titrated to effect compared to bolus injections [16]. For morphine for example, it has already been shown that with CRI compared to IM bolus administrations a smaller dose was necessary to achieve equal analgesic effects. Further, in horses adverse effects of butorphanol were reduced when administered via CRI compared to bolus injections [17]. Therefore, the primary aim of this study was to evaluate a methadone CRI at a preset dose regarding its anti-nociceptive properties in an acute pain model and associated adverse effects. A secondary aim was to collect pharmacokinetic data of this methadone CRI and to establish methadone plasma levels, corresponding to an anti-nociceptive effect in healthy beagle dogs.

## Results

### Nociceptive thresholds

**Fig. 1:**
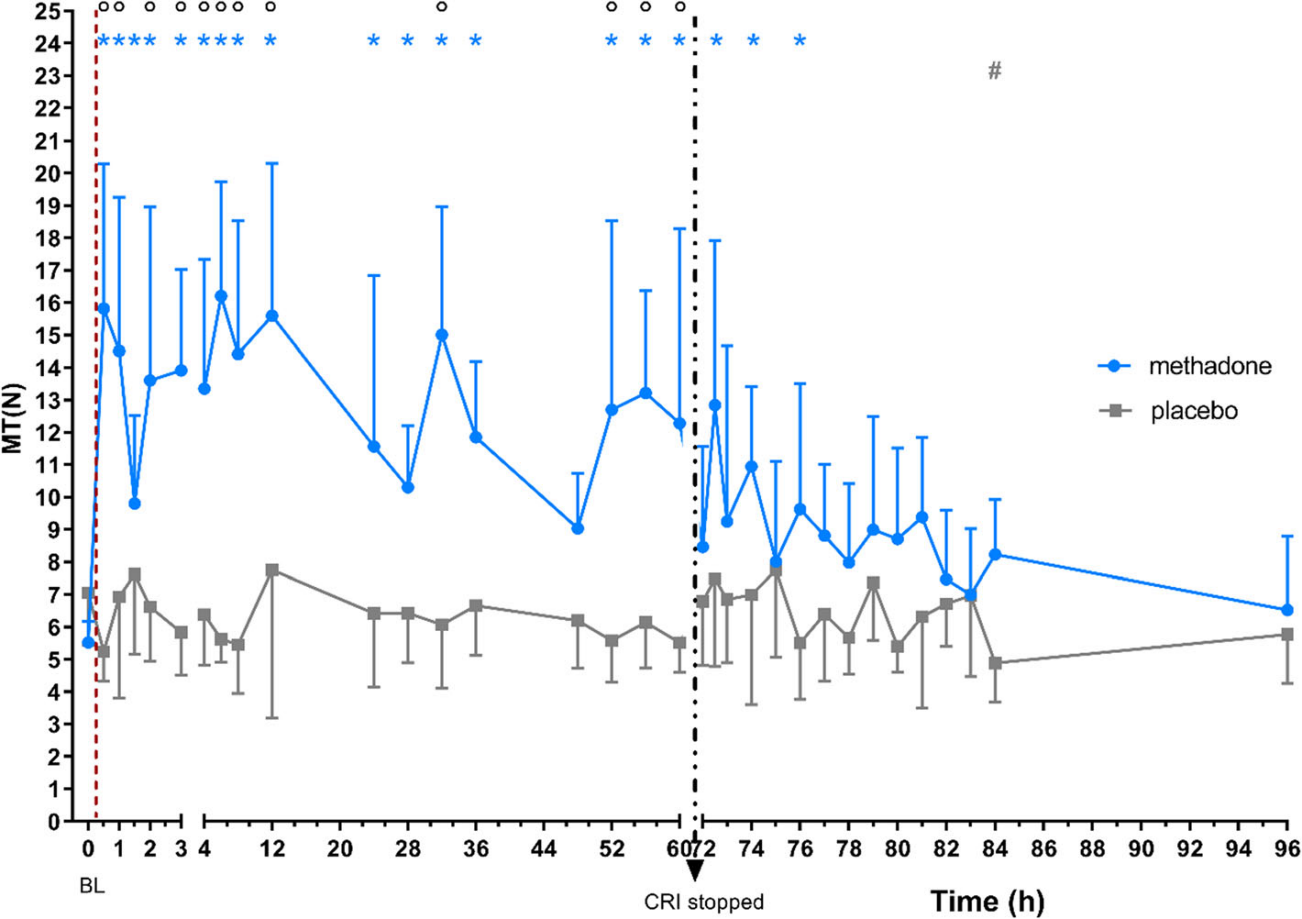
mean ± SD of mechanical threshold (MT) in N over time (*n* = 6); BL: baseline, dotted line: time of drug application; dotted arrow: stop of CRI; * significant difference (*p* < 0.05) to BL in group M; # significant difference (*p* < 0.05) to BL in group P; ° significant difference (*p* < 0.05) between groups

For MT only the data from six of seven dogs were included in the analysis due to technical problems with the measurements in one dog. In group M, MT was significantly higher compared to BL from 0.5 to 36 h, 52 to 60 h and at 72.5 h (*p* = 0.0004), 74 h (*p* = 0.0086) and 76 h (*p* = 0.0458). In group P, MT did not increase at any time point compared to BL, while at 96 h it was statistically lower than BL (*p* = 0.0330). Compared to placebo, MT was significantly higher in M from 0.5 to 12 h, at 32 h (*p* = 0.0001) and 52 to 60 h (Fig. 1).

**Fig. 2:**
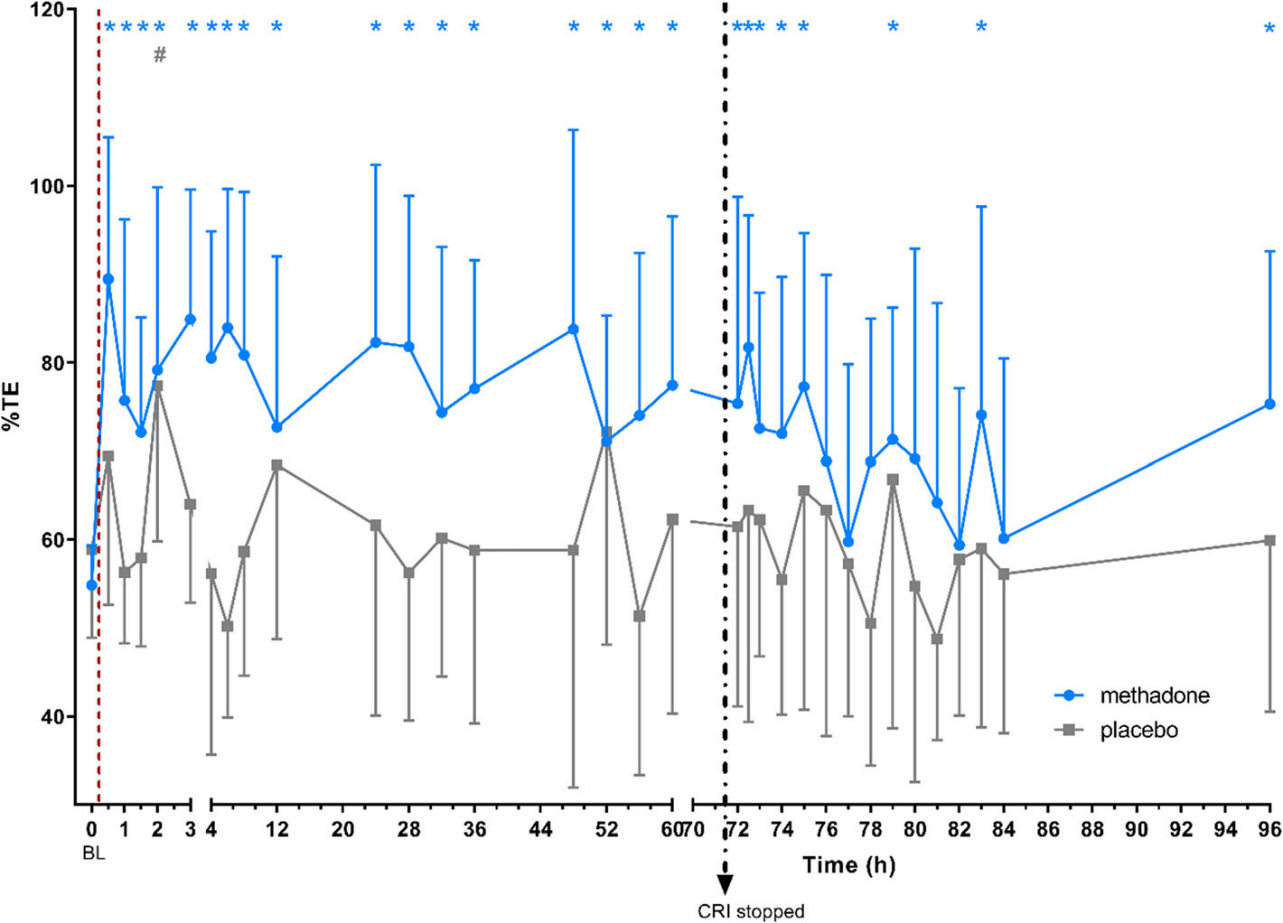
mean ± SD of thermal excursion (%TE) in % over time; BL: baseline; dotted line: time of drug application; dotted arrow: stop of CRI; * significant (*p* < 0.05) difference to BL in group M; # significant (*p* < 0.05) difference to BL in group P

In group M, percentage thermal excursion (%TE) was significantly increased from time point 0.5 to 75 h as well as at the time points 79 h (*p* = 0.0462), 83 h (*p* = 0.0202) and 96 h (*p* = 0.0135) compared to BL. In group P, a single significant increase in %TE from BL was detected at time point 2 h (*p* = 0.0150). Differences between treatments in %TE were not statistically significant at any time point (Fig. 2).

### Pharmacokinetics

The methadone plasma concentration-time profile best fitted a one-compartment model simulating infusion profile.

Main pharmacokinetic data are illustrated in Table 1. Steady state was estimated to occur after 12 h with average plasma levels of 29 to 35 ng ml^− 1^ and ranged from 23 to 49 ng ml^− 1^, respectively. The average coefficient of variation was 11.7%.

**Table 1:** Pharmacokinetic data of methadone CRI in mean ± SD

	Mean	SD
Total body clearance (ml kg^− 1^ min^− 1^)	51.44	9.47
Mean residence time (h)	3.40	1.74
Volume of distribution at steady state (l kg^− 1^)	10.26	5.53
Terminal half-life (h)	2.36	1.20

**Fig. 3:**
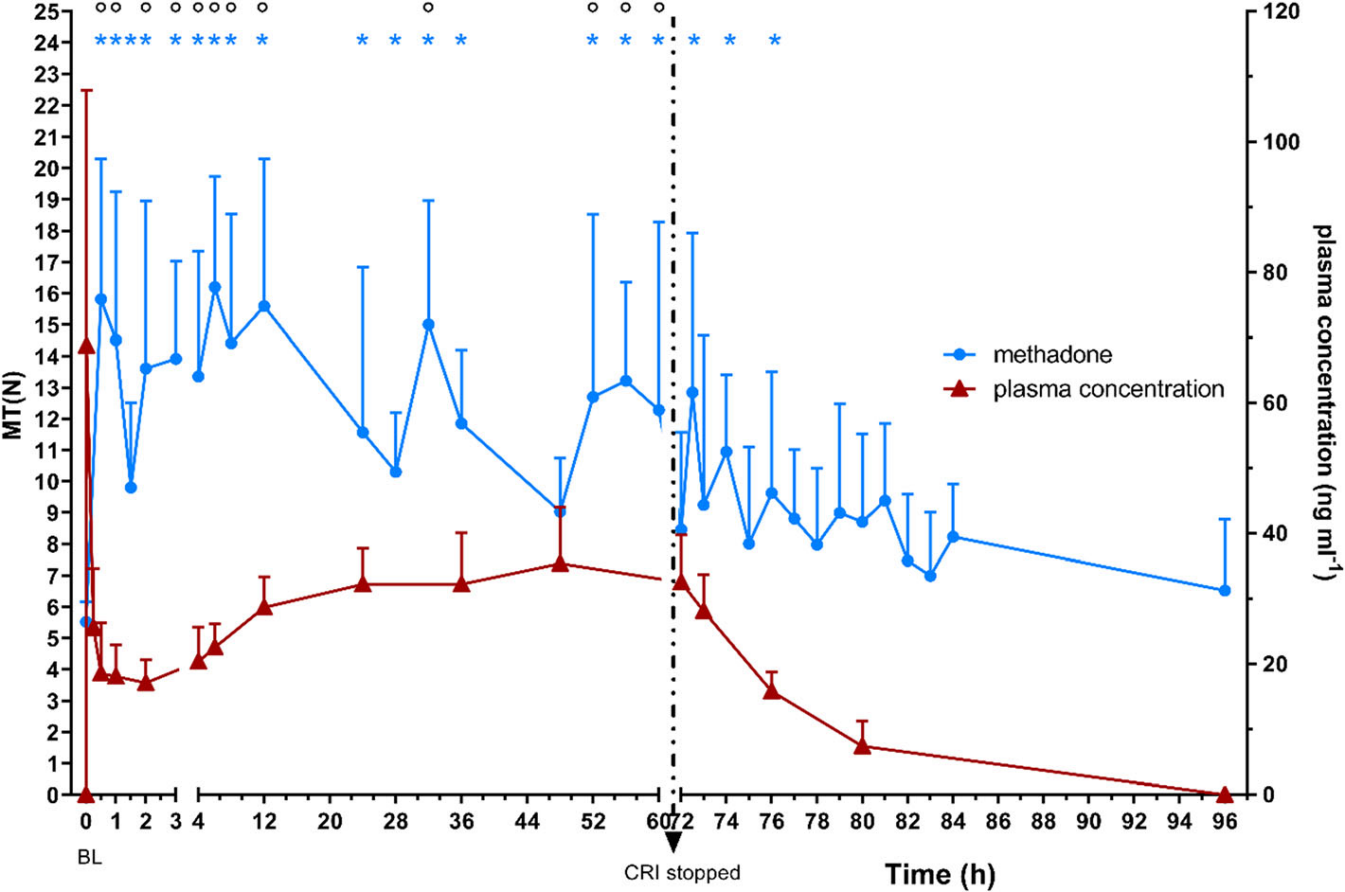
mechanical thresholds (MT) in N of group M over time with corresponding methadone plasma levels in ng ml^− 1^, both as mean ± SD; BL: baseline; dotted arrow: stop of CRI; * significant difference (*p* < 0.05) to BL in group M; ° significant difference (*p* < 0.05) between groups

**Fig. 4:**
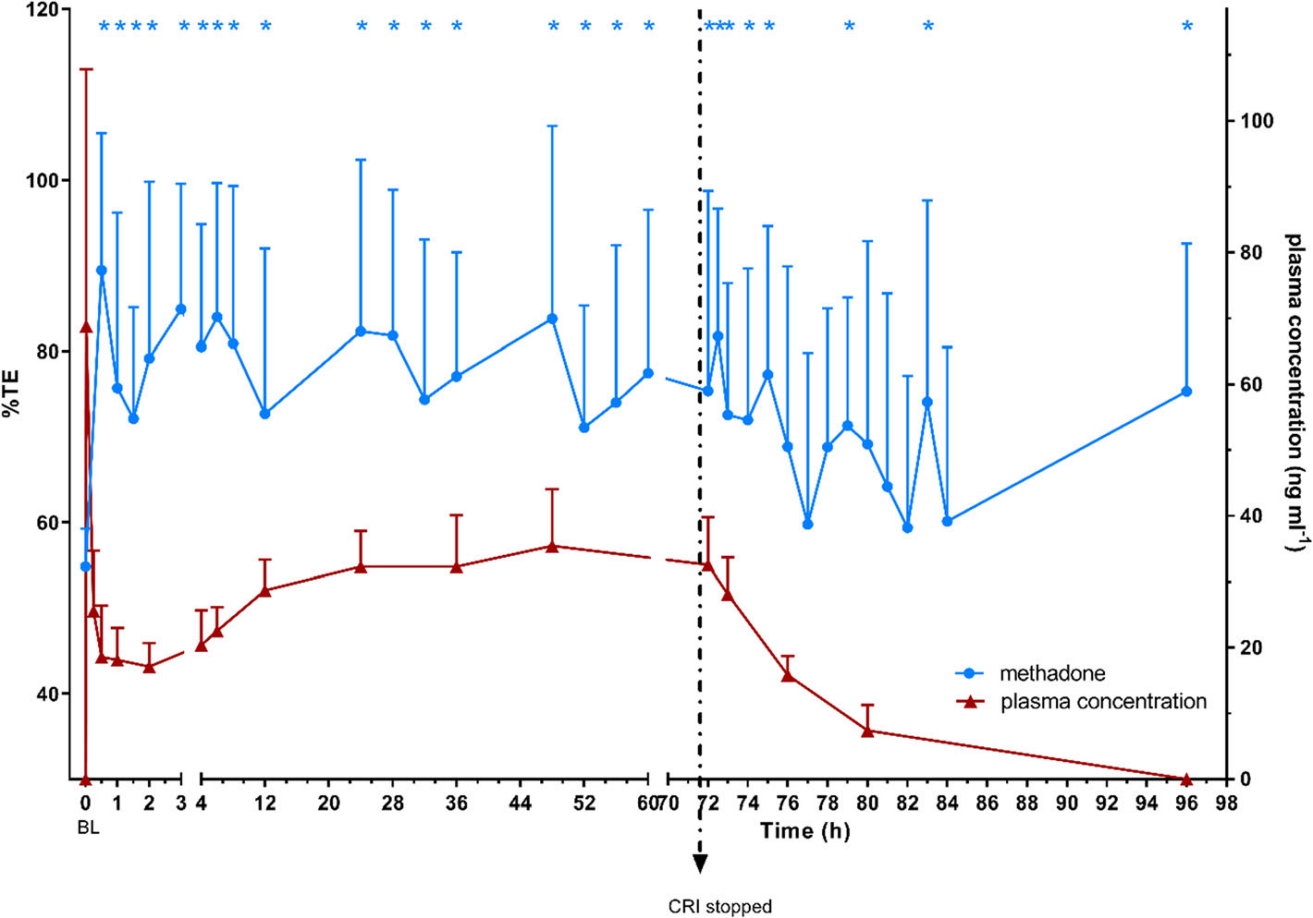
thermal excursion (%TE) in % of group M over time with corresponding methadone plasma levels in ng ml^− 1^, both as mean ± SD; BL: baseline; dotted arrow: stop of CRI; * significant (*p* < 0.05) difference to BL in group M

Nociceptive thresholds of treatment group M with corresponding plasma levels are illustrated in Figs. 3 and 4. Methadone plasma levels above 17 ng ml^− 1^ were associated with a significant increase in both nociceptive thresholds.

### Vital parameters

Respiratory rate (RR) significantly decreased over time in both treatment groups. Heart rate (HR) significantly decreased in treatment M from baseline and in comparison to P during administration of CRI and returned to BL values after completion of CRI (Table 2). Systolic, mean and diastolic blood pressures (SAP, MAP, DAP) were not altered over time in both treatment groups, rectal temperature (Temp) decreased significantly in treatment M from baseline and in comparison to P during CRI and returned to BL values after completion of CRI (Table 3).

**Table 2:** Mean ± SD heart rate and respiratory rate in both groups at different time points

Time (h)	Heart rate (beats min^−1^)		Respiratory rate (breath min^− 1^)	
	Group M	Group P	Group M	Group P
BL	87 ± 12	82 ± 11	16 ± 3	19 ± 4
0.75	66 ± 17*	83 ± 9	12 ± 2*	15 ± 5*
1.75	59 ± 13*	82 ± 14	12 ± 2*	14 ± 3*
2.75	63 ± 13*° (*p* = 0.0007)	94 ± 8*	13 ± 2*	18 ± 3
3.75	65 ± 11*	89 ± 6	13 ± 2*	14 ± 5*
5.75	61 ± 10*	79 ± 15	13 ± 2*	14 ± 5*
7.75	59 ± 13*	84 ± 7	12 ± 1*	14 ± 4*
9.75	59 ± 10*	82 ± 12	14 ± 2	14 ± 6*
11.75	57 ± 7*° (*p* = 0.0027)	86 ±10	13 ± 2*	13 ± 3*
23.75	66 ± 18*° (*p* = 0.0127)	93 ± 7*	14 ± 2	17 ± 3
25.75	55 ± 13*° (*p* = 0.0002)	88 ± 16	12 ± 0*	13 ± 2*
27.75	61 ± 14*	85 ± 14	15 ± 4	13 ± 2*
29.75	57 ± 12*° (*p* = 0.0072)	85 ± 16	11 ± 2*	14 ± 3*
31.75	59 ± 12*° (*p* = 0.0014)	89 ± 10	12 ± 0*	13 ± 3*
33.75	62 ± 14*	85 ± 10	11 ± 1*	12 ± 1*
35.75	60 ± 12*° (*p* = 0.0011)	90 ± 9	12 ± 0*	15 ± 3*
47.75	65 ± 16*° (*p* = 0.0182)	91 ± 9	13 ± 2*	16 ± 2
49.75	63 ± 18*	85 ± 10	12 ± 0*	14 ± 2*
51.75	63 ± 13*	83 ± 12	11 ± 1*	15 ± 4*
53.75	62 ± 14*° (*p* = 0.0260)	88 ± 7	12 ± 2*	13 ± 3*
55.75	55 ± 5*° (*p* = 0.0182)	82 ± 17	12 ± 0*	13 ± 2*
57.75	63 ± 14*	85 ± 12	12 ± 0*	13 ±2*
59.75	63 ± 11*° (*p* = 0.0018)	93 ± 8	13 ± 2*	14 ± 2*
71.75	69 ± 16*	87 ± 18	14 ± 3	14 ± 3*
73.75	71 ± 17*	86 ± 14	13 ± 2*	14 ± 2*
75.75	75 ± 28*	87 ± 16	13 ± 2*	13 ± 2*
77.75	80 ± 28	82 ± 18	15 ± 4	12 ± 3*
79.75	95 ± 27	88 ± 12	14 ± 4	13 ± 2*
81.75	90 ± 16	78 ± 15	14 ± 2	13 ± 2*
83.75	103 ± 13*	93 ± 16*	15 ± 3	14 ± 2*
95.75	103 ± 14*	89 ± 9	17 ± 5	17 ± 3

*BL* Baseline

*significant difference (*p* < 0.05) to baseline in M

° significant difference (*p* < 0.05) between groups, displayed *p* values refer to significant differences between groups

**Table 3:** Mean ± SD systolic, mean an diastolic arterial pressure (SAP, MAP, DAP) in mmHg and temperature in °C of both groups at different time points

**Parameter**	**Group**	**BL**	**0.75 h**	**1.75 h**	**3.75 h**	**7.75 h**	**11.75 h**	**23.75 h**	**27.75 h**	**31.75 h**	**35.75 h**
SAP (mmHg)	M	140 ± 16	139 ± 7	141 ± 15	146 ± 14	147 ± 13	154 ± 7*	147 ± 13	149 ± 8	150 ± 9	152 ± 10*
	P	142 ± 18	139 ± 12	140 ± 19	140 ± 12	144 ± 15	141 ± 10	137 ± 17	142 ± 10	147 ± 11	140 ± 9
MAP (mmHg)	M	104 ± 9	98 ± 10	104 ± 8	103 ± 12	102 ± 13	108 ± 7	106 ± 12	103 ± 9	104 ± 8	106 ± 11
	P	102 ± 13	102 ± 10	100 ± 9	103 ± 10	104 ± 11	100 ± 5	103 ± 13	86 ± 8	107 ± 7	103 ± 8
DAP (mmHg)	M	85 ± 8	79 ± 11	86 ± 10	81 ± 11	81 ± 15	85 ± 9	86 ± 12	83 ± 12	80 ± 7	86 ± 11
	P	84 ± 11	83 ± 13	80 ± 9	82 ± 5	84 ± 12	83 ± 4	82 ± 8	106 ± 8	85 ± 9	86 ± 7
Temperature (°C)	M	38.4 ± 0.1	37.1 ± 0.4*° *p* = 0.0002	36.7 ± 0.5*° *p* < 0.0001	36.7 ± 0.4*° *p* < 0.0001	36.5 ± 0.4*° *p* < 0.0001	36.9 ± 0.3*° *p* < 0.0001	37.0 ± 0.3*° *p* < 0.0001	36.5 ± 0.4*° *p* < 0.0001	36.7 ± 0.2*° *p* < 0.0001	36.9 ± 0.4*° *p* < 0.0001
	P	38.4 ± 0.4	38.1 ± 0.3	38.1 ± 0.3	38.0 ± 0.1*	37.9 ± 0.3*	38.3 ± 0.3	38.6 ± 0.3	37.9 ± 0.1*	38.0 ± 0.3*	38.4 ± 0.3
**Parameter**	**Group**	**47.75 h**	**51.75 h**	**55.75 h**	**59.75 h**	**71.75 h**	**75.75 h**	**79.75 h**	**83.75 h**	**95.75 h**	
SAP (mmHg)	M	148 ± 13	146 ± 14	149 ± 16	154 ± 13*	146 ± 15	145 ± 16	144 ± 12	143 ± 6	145 ± 8	
	P	148 ± 18	141 ± 10	146 ± 11	144 ± 17	142 ± 7	141 ± 13	141 ± 11	145 ± 11	145 ± 18	
MAP (mmHg)	M	105 ± 9	101 ± 9	107 ± 8	107 ± 9	102 ± 14	106 ± 10	108 ± 9	110 ± 4	104 ± 5	
	P	107 ± 17	105 ± 10	107 ± 6	108 ± 11	105 ± 4	106 ± 13	103 ± 8	109 ± 7	103 ± 13	
DAP (mmHg)	M	85 ± 10	80 ± 10	88 ± 8	87 ± 11	82 ± 13	86 ± 7	92 ± 8	89 ± 6	84 ± 6	
	P	87 ± 17	90 ± 11	88 ± 5	91 ± 11	86 ± 8	88 ± 13	83 ± 7	93 ± 6	81 ± 12	
Temperature (°C)	M	37.3 ± 0.4*° *p* < 0.0001	36.8 ± 0.5*° *p* < 0.0001	36.9 ± 0.4*° *p* < 0.0001	37.2 ± 0.4*° *p* < 0.0001	37.5 ± 0.6*° *p* < 0.0001	38.2 ± 0.4	38.6 ± 0.2	39.0 ± 0.3*	38.9 ± 0.2*	
	P	38.6 ± 0.5	38.2 ± 0.7	38.4 ± 0.6	38.5 ± 0.4	38.7 ± 0.4	38.3 ± 0.5	38.2 ± 0.3	38.4 ± 0.3	38.6 ± 0.4	

*BL* Baseline

*significant difference (*p* < 0.05) to BL

° significant difference (*p* < 0.05) between groups, displayed *p*- values refer to significant differences between groups

### Behaviour score

**Fig. 5:**
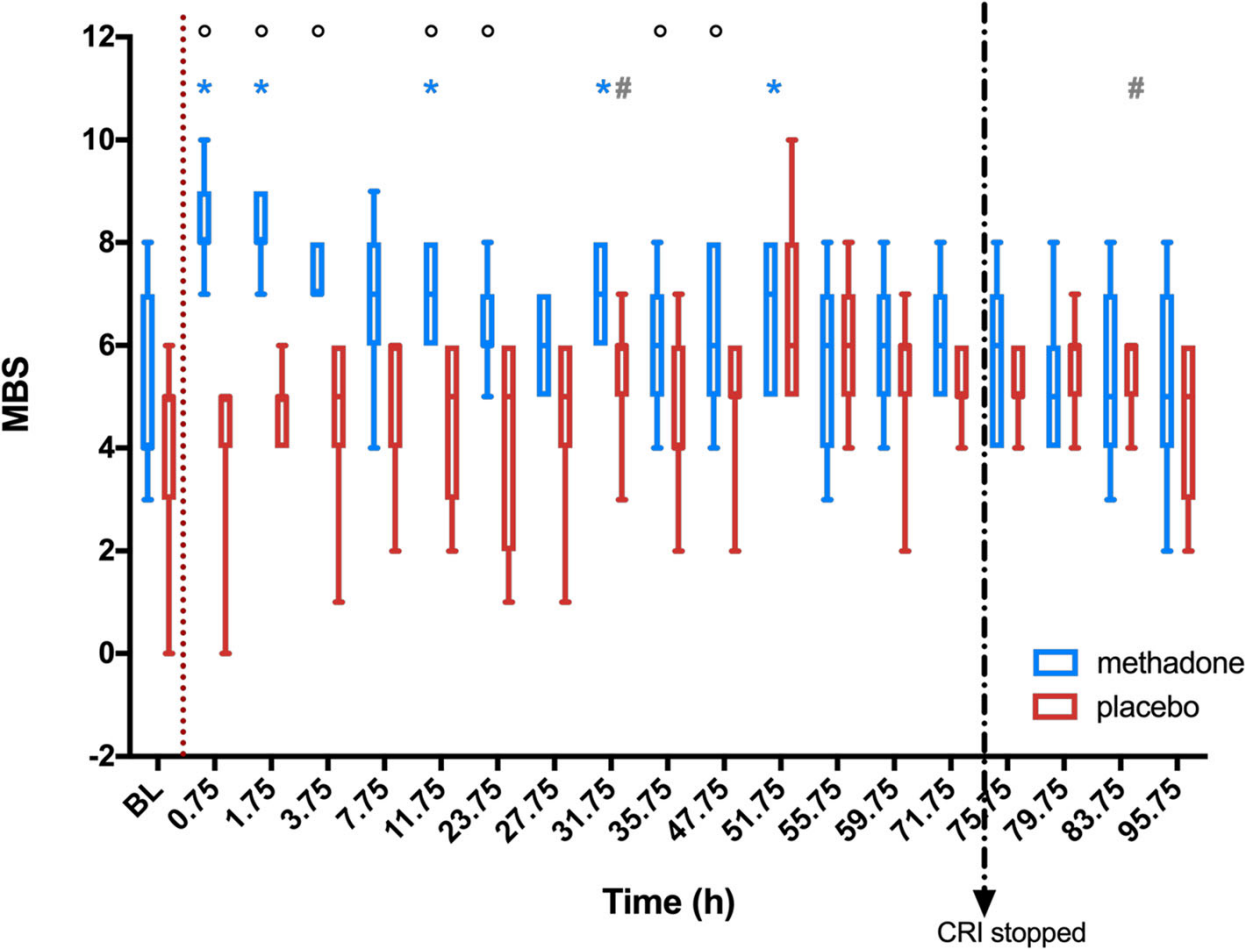
Sedation score over time as boxplots, with median (central line), inter-quartile range (box boundaries) and minimum and maximum (whiskers); dotted line: time of drug application; dotted arrow: stop of CRI; * significant difference (*p* < 0.05) to baseline in group M; # significant difference (*p* < 0.05) to baseline in group P, ° significant difference (*p* < 0.05) between groups

The multimodal behaviour score (MBS) was elevated from BL in treatment M at time points 0.75 h (*p* = 0.0156), 1.75 h (*p* = 0.0156), 11.75 h (*p* = 0.0313), 31.75 h (*p* = 0.0313), 51.75 h (*p* = 0.0313), and in group P at time points 31.75 h (*p* = 0.0313) and 95.75 h (*p* = 0.0313), respectively. Between both treatments MBS differed significantly at time points 0.75 to 3.75 h, 11.75 h (*p* = 0.0156), 23.75 h (*p* = 0.0313), 35.75 h (*p* = 0.0313) and 47.75 h (*p* = 0.0313) (Fig. 5).

### Gastrointestinal passage time (GIPT)

The GIPT did not differ between treatments (Table 4). GIPT could not be evaluated in one dog due to inappetence in group M (*n* = 6).

**Table 4:** Gastrointestinal passage time (GIPT) in h for both treatment groups in median with minimum and maximum in brackets

	Group M	Group P
GIPT (h)	28:18:45 [16:35:00; 55:06:00]	29:00:45 [27:51:30; 40:56:00]

### Further adverse effects

During methadone CRI administration 5 of 7 dogs showed decreased food intake, ranging from a not fully eaten half-daily ratio to inappetence for 47 h.

Furthermore, four animals regurgitated 2 to 12 times during CRI of methadone between 11 and 72.5 h after the start of CRI.

## Discussion

In the current study, the methadone CRI at a rate of 0.1 mg kg^− 1^ h^− 1^ after a loading dose produced consistent mechanical and thermal anti-nociceptive effects over 72 h infusion time without signs of significant tachyphylaxis or tolerance in an acute pain model in healthy beagle dogs. Typical opioid adverse effects like sedation, bradycardia and hypothermia were seen and, interestingly, some more uncommon gastrointestinal adverse effects like regurgitation and inappetence were observed. The investigated CRI dose was chosen based on two different bolus pharmacokinetic studies. In the first study, beagle dogs and beagle cross breeds received 0.4 mg kg^− 1^ methadone IV. In the second study plasma levels were determined in beagle dogs after a 0.2 mg kg^− 1^ IM bolus of levomethadone. The later study also investigated at which plasma levels an antinociceptive effect was observed [18, 19]. Plasma levels of 22.6–46.3 ng ml^− 1^ levomethadone revealed to be analgesic. Due to equipotency of levomethadone:methadone at a dose ratio of 1:2 in dogs [20, 21], plasma levels of 60 ng ml^− 1^ were targeted for racemic methadone for dose calculation in our study.

In the present study two different nociceptive modalities have been used to test the efficacy of the 0.1 mg kg^− 1^ h^− 1^ methadone CRI. The combination of mechanical and thermal threshold tests provided valid and reproducible data in various studies investigating anti-nociceptive properties of opioids in small animals [18, 22–26]. Further on, μ-agonistic opioids are preferentially inhibiting C-fibres [27] and the rates of heat and pressure increases used in this study are suitable for stimulation of C-fibres [28, 29] Significant increases in mechanical and thermal thresholds from baseline in treatment group M as well as a significant difference in mechanical thresholds between treatment groups can be assumed as indicative of an anti-nociceptive effect of the methadone CRI. However, the observed group difference in thermal threshold and %TE was not statistically significant. This might be partially explained by the low cut-out temperature, set at 50 °C. While in comparable studies cut-out temperatures of up to 55 °C were chosen, mild skin burns in a pilot trial in our beagle dogs led us to a stepwise reduction of the maximal temperature. Consecutively further significant differences might have been masked as differentiation was less. Furthermore, the variance was comparatively high, contributing to the fact that the difference between treatments did not gain statistical significance. However, the amount of the thermal threshold increase from baseline after treatment with methadone was of statistical and clinical significance. Nevertheless, it is important to note that cutaneous antinociception as tested in this experimental study setting is not the same as a complex clinical pain syndrome. Thermal and mechanical threshold testing is an experimental option to test antinociceptive effectiveness of opioids, treatment of severe pain states however might necessitate higher doses or additional analgetic drugs acting at different sites of the pain pathway.

When our results of anti-nociceptive testing are set in relation to the measured methadone plasma concentrations, the plasma concentrations associated with thermal and mechanical anti-nociception in the acute pain models were above 17.14 ng ml^− 1^. The only study investigating the antinociceptive effects of methadone in relation to plasma levels in dogs used levomethadone in fixed combination with fenpipramide. In that study, thermal and mechanical thresholds and corresponding plasma concentrations after IM bolus injection of 0.2 mg kg^− 1^ of levomethadone were measured; plasma concentrations ranging between 22.6 and 46.3 ng ml^− 1^ were effective [18]. As levomethadone is the active enantiomer at the μ-receptor, equipotency with the racemate is generally estimated at dose ratios of 1:2 [20, 21]. This has been proven in beagle dogs in a thermal and mechanical threshold testing model at doses of 0.2 mg kg^− 1^ and 0.4 mg kg-^1^ of levomethadone and racemic methadone, respectively [21]. Based on this 1:2 dose ratio for equipotency antinociceptive plasma levels were more than twice as high in the study by Hoffman et al. [18] compared to our study. However, different application routes were used in the two studies (IM vs IV), as well as different drug formulations. It is unclear, if the fixed combination with the anticholinergic fenpipramide could have had any impact on the results in that study. In addition, plasma levels are commonly used to describe dose-effect-relations but do not reflect true opioid concentrations or receptor binding, respectively. Furthermore, plasma levels of methadone were not determined for each time point of nociceptive testing in this study.

The aim of administering methadone as CRI was to reduce the dose-dependent adverse effects that were seen after bolus administration. However, a significant adverse effect observed in this study was hypothermia, with the profoundest reduction in body temperature of 1.9 °C at 7.75 h and 27.75 h. Hypothermia is a common adverse effect of μ-agonists in dogs, caused by direct effects on the thermoregulation centre and by reduction of the metabolic rate [30, 31]. Bolus injection of 0.5 mg kg^− 1^ methadone IM caused an average reduction of rectal temperature of 2.2 °C in dogs [13]. A second study in dogs revealed that this effect is dose-dependent: Bolus injection of 0.3 mg kg^− 1^ methadone IM caused a reduction of 0.7 °C, 0.5 mg kg^− 1^ IM a reduction of 1.7 °C and 1.0 mg kg^− 1^ IM a reduction of 2.3 °C, respectively [15]. However, the application as CRI in this study resulted in a comparable degree of hypothermia and not in a significant reduction of this adverse effect.

Hypothermia consecutively resulted in a reduction in skin temperature in this study. Therefore, %TE was calculated and analysed instead of simple TT values to avoid this as a potential bias. Interestingly a circadian rhythmicity of body temperature which is described elsewhere in dogs is still notable under methadone CRI [32].

A second important side effect observed in this study was the decrease in HR. We hypothesized that this would be less pronounced with the CRI administration, however, heart rate decreased by 21–37% compared to BL during the CRI. A reduction in HR is a common side effect of μ-agonists in dogs [11, 13, 15, 33] and originates mainly from an increase in vagal tone [10, 34–36]. It is controversial if this effect is dose-dependent. After IM bolus administration of 3 different doses of methadone (0.3; 0.5; 1.0 mg kg^− 1^), no clear dose dependence was revealed with a decrease in HR of 13–27%, 5–22% and 7–20%, respectively [15]. At higher doses and IV application, however, reduction of heart rate was dose dependant with 19–28% after 0.5 mg kg^− 1^ IV and 32–46% after 1.0 mg kg^− 1^ IV [12]. The drop in heart rate we observed is comparable to a decrease described after IM bolus injection of 0.5 mg kg^− 1^ in dogs [15], therefore at the investigated dose no advantage of the CRI application was proven concerning this adverse effect.

Concurrent with bradycardia (and sedation), a consecutive drop in blood pressure is usually expected due to the reduction in cardiac output. Interestingly, blood pressure was not altered in this study under methadone CRI. One possible explanation for this might be a rise in vasopressin plasma levels, which had been observed after methadone bolus administration at doses of 0.4–1 mg kg^− 1^ [11, 19, 33].

A dose dependent mild to moderate sedation had been identified in various studies in dogs receiving methadone and was also obvious in this study [12–15]. Interestingly, the sedative effect diminished during the CRI administration. A possible explanation might be the development of early tolerance, leading to termination of the sedative effect before the end of methadone exposure. An effect due to the initial bolus administration might have played a role, but is less likely, because plasma concentrations were steady for 35 h prior to termination of sedative effect. Furthermore, a light tendency to decreased mechanical thresholds under steady state of methadone CRI was observed, which might corroborate the theory of initialization of tolerance. Further studies with longer methadone CRI are needed to fully estimate the possibility and degree of development of tolerance.

The μ-opioids lead to a reduction in respiratory rate and tidal volume up to apnea in a dose dependent manner [37]. In this study a reduction in respiratory rate was observed over time without a group difference. So, no effect of methadone on respiratory rate was detected, which might have resulted from the relatively small dosage or could be an advantage of the application route as a CRI. An effect on tidal volume cannot be ruled out in this study design. The difference in respiratory rate in both groups to baseline could be due to calming.

All in all the observed adverse effects might be less in animals suffering pain than in this experimental trial in healthy dogs [38]. For example panting is much more common in dogs, which are not in pain [39].

Further described dose dependent adverse effects after methadone bolus administration like salivation, defecation, vocalization or dysphoric behaviour could not be seen in this study [11–15, 19]. However, obvious gastrointestinal adverse effects occurred like inappetence, which could be due to the significant sedative effect of the methadone CRI, as with diminishing sedation more and more food was eaten. In addition, slowed gastric emptying, which is known to be caused by administration of μ-agonists could have contributed to the decrease in food intake [40]. In one study, electrically induced inhibition of gastric motility and emptying in dogs let to a significant reduction in food intake [41]. Nausea could be another reason for inappetence, but no additional clinical signs like hypersalivation, gagging, licking or vomitus were recorded. Furthermore, methadone is comparatively less likely to cause nausea than more hydrophilic opioids [42], but nausea cannot entirely be ruled out in this study design.

Second, four dogs showed passive reflux, without any signs of gagging or contractions of the abdominal muscles. It was mostly associated with standing up out of a lying position. Therefore, in the opinion of the observing authors it was judged to be regurgitation. This phenomenon has not yet been described for methadone to the author’s knowledge; in contrast methadone is known to be antiemetic at higher dosages [42]. A possible explanation could be again the inhibitory effect of μ-agonists on gastric emptying and decreased motility and further on a decreased lower oesophageal sphincter tone [40, 43]. These effects are also discussed in humans as a cause of the higher risk of regurgitation with the use of opioids in the perioperative setting [44].

Constipation is another common adverse effect of opioids [45]. This could not be detected in our study, however, the inappetence of some animals prevented the sufficient uptake of charcoal, thereby reducing the sensitivity of this analysis.

An estimated steady state of methadone plasma levels was reached after 12 h, with a relatively low coefficient of variation of 11.7% and no evidence of further accumulation. Volume of distribution (V_d_) was high in this study and similar to the V_d_ described after bolus injection of 0.4 mg kg^− 1^ methadone IV [19]. The similar V_d_’s after bolus injection or after CRI with initial bolus injection further support the assumption that no accumulation occurred after reaching the plateau after 12 h. However, a difference between the volume of distribution of a bolus injection and of a CRI, administered over a longer period, can occur. The Clearance (Cl) of methadone after bolus injection of 1 mg kg^− 1^ and 0.4 mg kg^− 1^ has been reported to be high with 25.4 ml kg^− 1^ min^− 1^ and 27.9 ml kg^− 1^ min^− 1^_,_ respectively [19, 46]. In the present study, however, Cl was twice as high and therefore comparable to the Cl reported in greyhounds after bolus injection of 0.5 mg kg^− 1^ IV (56.04 ml kg^− 1^ min^− 1^) [47]. Methadone predominantly is metabolized by the liver and biliary excreted, with a minor part being metabolized by the kidneys. As methadone is metabolized by the cytochrome p450 system, breed differences and genetic variation may play an important role when comparing the Cl in different studies [46]. Less is unchanged excreted with urine and faeces [48]. So metabolization of methadone depends on renal and hepatic blood flow, which is described in dogs as 21.6 ml kg^− 1^ min^− 1^ and 30.9 ml kg^− 1^ min^− 1^, respectively [49]. Those two blood flow rates match the detected clearance, therefore an extrahepatic metabolization seems to be unlikely. This is in accordance with the pharmacokinetic data observed after bolus injection in beagle and beagle mix breeds [19, 46].

Terminal half-life (t_0.5_) depends on Cl and V_d_ as t_0.5_ = 0.693 V_d_ Cl^− 1^. As V_d,_ as well as Cl, were higher in this study, t_0.5_ was similar to t_0.5_ observed after bolus injection. After 1.0 mg kg^− 1^ methadone IV t_0.5_ was 1.75 h whereas after 0.4 mg kg^− 1^ methadone IV a t_0.5_ of 3.9 h is described in beagle dogs and beagle mixed breeds [19, 46]. In this study a t_0.5_ of 2.4 h was detected, which is 35% longer and 39% shorter than the two described after bolus injection, respectively. A possibly influencing factor could be the duration of measurement after drug administration, with results being more accurate after a longer measurement period [19]. Ingvast-Larsson et al. measured for 24 to 30 h after bolus injection whereas Kukanich et al. only measured for 8 h after administration. In this study after stop of CRI, plasma levels were measured for 24 more hours. Further influencing factors could have been the different methods to determine plasma levels of methadone and the different limits of quantification (LOQ), with a more accurate result with a lower limit of quantification. In the study of Ingvast-Larsson et al. a liquid chromatography-electrospray ionization-tandem mass spectroscopy with a LOQ of 0.6 ng ml^− 1^ was used, whereas Kukanich et al. used a high-pressure liquid chromatography with ultraviolet detection and a LOQ of 20 ng ml^− 1^ or a fluorescence polarization immunoassay with a LOQ of 25 ng ml^− 1^, respectively. In this study gaschromatography-massspectroscopy (GC-MS) was used with a LOQ of 5 ng ml^− 1^. But, overall an obvious change in context sensitive half-life like it is described for fentanyl CRI in dogs [50] seems unlikely based on the available dog data. But this hypothesis is only based on comparison to other studies. To verify this hypothesis a direct comparison between pharmacokinetics after bolus injection and CRI in the same dogs are needed. Furthermore, a more frequent measurement of plasma levels after discontinuation of methadone CRI is recommended to exclude the possibility of underestimation of t_0.5_.

## Conclusion

A methadone bolus followed by a CRI administered at a rate of 0.1 mg kg^− 1^ h^− 1^ for 3 days leads to cutaneous antinociception, while it doesn’t lead to obvious signs of tolerance or tachyphylaxis. Methadone plasma concentrations above 17.1 ng ml^− 1^ are associated with acute antinociception. Pharmacokinetic data reveal that a steady state is reached after 12 h with no signs of accumulation. Typical adverse effects that are described after methadone bolus administration such as hypothermia, bradycardia and sedation are also observed with the CRI administration in healthy dogs.

Interestingly, gastrointestinal signs like regurgitation and inappetence are observed in this experimental study. Further studies investigating the advantages of a methadone CRI in the treatment of clinical pain are needed.

## Methods

### Study design and animals

The study was performed at the Small Animal Clinic of the University of Veterinary Medicine Hannover, Foundation. It was approved by the ethical committee of the Lower Saxony State Office for Consumer Protection and Food Safety according to the german animal welfare act (approval number 33.12–42,502–04-14/1733). Seven purpose-bred (at our own institution) adult beagle dogs, 2 spayed females and 5 neutered males, aged between 47 and 54 months and weighing between 9.8 and 21.2 kg were included in this study. All dogs were healthy based on clinical examination, blood cell count and serum biochemistry. Each dog received two treatments in a blinded randomized complete cross-over design with a washout period of at least 14 days between treatments. Randomisation was performed using an open accessed randomizer (www.randomizer.org). Dogs of the M group were administered a bolus of 0.2 mg kg^− 1^ methadone IV directly followed by a CRI of 0.1 mg kg^− 1^ h^− 1^ IV for 72 h. Dogs of the P group were administered a bolus injection and a CRI IV of an equivalent volume of isotonic saline solution for 72 h. Dosage of methadone CRI was calculated based on methadone bolus pharmacokinetic data [19] and antinociceptive plasma levels after a levomethadone bolus IM [18]. During the trial, each dog was held in a separate playpen with a familiar dog in direct contact in a second playpen in an examination room. Dogs were fed twice daily with commercial dry food and had free access to water. Overnight they were held in two cages next to each other in the clinic section. In between the trial days dogs were assessed healthy based on a clinical examination, complete blood count and biochemistry and were reintegrated in their groups. After completing the trials dogs were again assessed healthy based on clinical and blood parameters and were reintegrated in their groups.

All dogs were held in groups of 5–7 animals per group with daily periods in enriched run areas outside and walks with veterinary students.

### Instrumentation

**Fig. 6:**
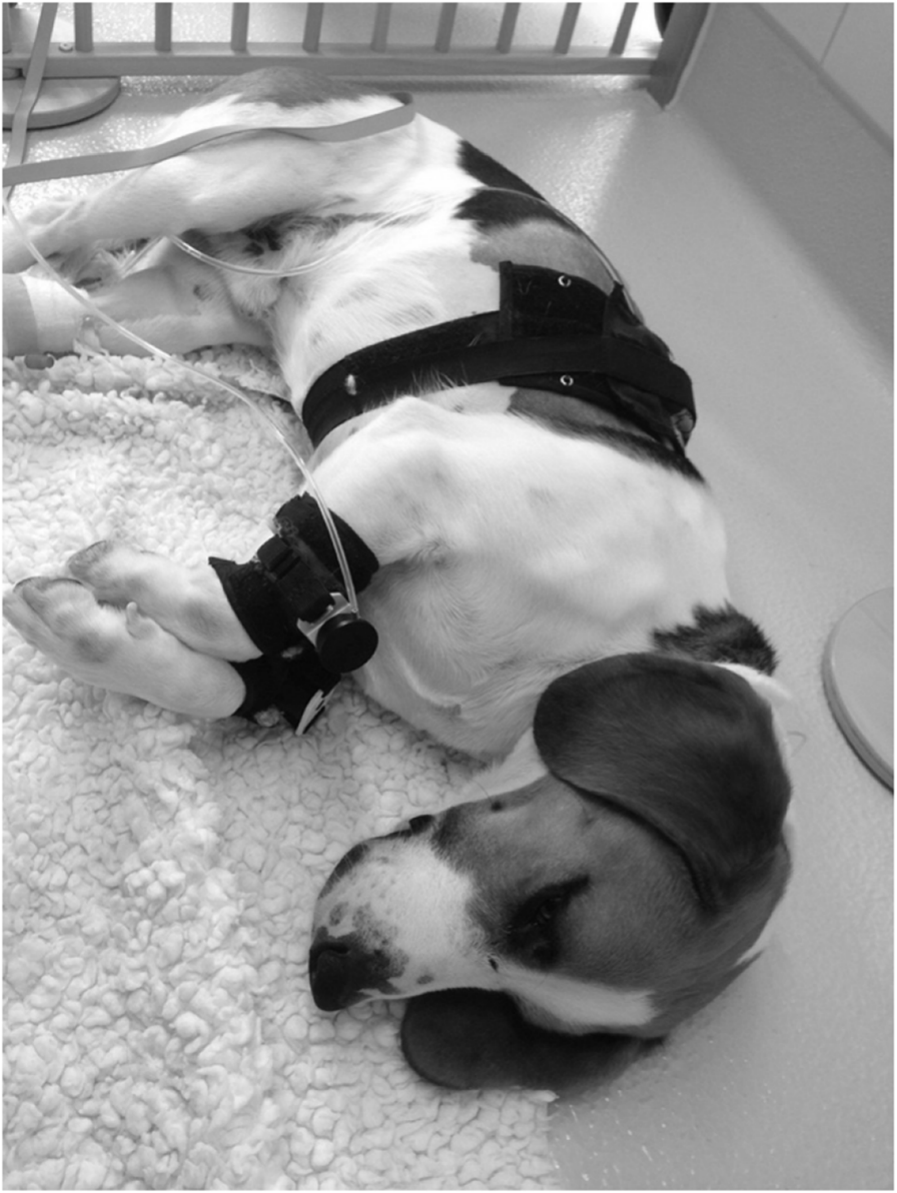
An instrumented dog relaxing during testing between applications of the stimuli in the play pen. The thermistor probe for thermal testing is held against the thoracic wall with elastic ribbons. The mechanical probe is placed above radius and ulna of the right forelimb and a dummy on the contralateral foreleg

Dogs were trained to wear the stimulation probes for several weeks prior to the study to minimize stress and adverse behaviour. One day before the start of the measurements, dogs were instrumented with a central venous catheter in a jugular vein for drug administration and a peripherally inserted catheter in a femoral vein for blood sampling. At the days of the trials, beagles were instrumented with a mechanical probe, containing a single metal pin with a flat tip, 2 mm in diameter over the dorsal aspect of the right or left radius and ulna. A non-functional dummy was placed over the dorsal aspect of radius and ulna of the contralateral side. They were connected to a wired mechanical threshold testing device (MT1, Topcat Metrology Ltd., UK), which had been evaluated for use in dogs [51]. For thermal threshold testing a thermode based system was used (TT1, Topcat Metrology Ltd., UK). Its thermistor probe was placed on the previously clipped area of the lateral thoracic wall and secured with elastic ribbons. It was connected to the control unit and a bladder behind the thermistor probe was filled with air until a pressure between 30 and 80 mmHg was reached to guarantee constant skin contact (Fig. 6).

### Measurements

A single investigator blinded to the treatment groups until completion of trials performed all measurements.

#### Nociceptive stimulation

Measurements started after an equilibration time of 30 min after instrumentation with the stimulation systems to allow the dogs to adapt. First three baseline measurements were performed for both stimulation systems, which were averaged afterwards. After administration of drug or placebo further stimulations were performed at 0.5, 1, 1.5, 2, 3, 4, 6, 8, 12, 24, 28, 32, 36, 48, 52, 56, 60, 72, 72.5, 73, 74, 75, 76, 77, 78, 79, 80, 81, 82, 83, 84 and 96 h after administration.

For mechanical stimulation the mechanical probe was driven by a rolling diaphragm, so that a single pin with a flat 2 mm in diameter tip was pressed against the forelimb, with the force being applied manually via a syringe. Force was continuously increased with 0.8 Newton (N) second^− 1^, controlled by signal lights, until the dogs showed a defined directed reaction (turning head to stimulated leg, vocalizing, leg lifting, licking) or until a safety cut-out of 20 N was reached.

For thermal threshold testing, first the skin temperature was recorded. Afterwards heating was started with a temperature increase of 0.6 °C second^− 1^ until dogs showed a defined directed reaction (turning head to stimulation side, skin twitch, vocalization, scratching with legs, repositioning or licking) or the safety cut-out of 50 °C was reached. To exclude possible influences of variable skin temperatures, %TE was calculated as follows including skin temperature (T_S_), reaction temperature (T_R_) and cut-out temperature (T_C_) in the equation below [52]:

$$ \% TE=100\times \frac{\left(\mathrm{TR}-\mathrm{TS}\right)}{\left(\mathrm{TC}-\mathrm{TS}\right)} $$

Mechanical stimulation was always performed prior to thermal stimulation and dogs were de-instrumented from stimulation systems, when time between measurements was more than 30 min or overnight and re-instrumented 30 min before the next stimulation and at the next morning, respectively.

#### Vital parameter

The HR, RR, arterial blood pressures (SAP, DAP, MAP) and rectal Temp were measured using auscultation, chest movements, an oscillometric device (Petmap®) with the cuff over the coccygeal artery and a commercially available digital thermometer (VT 1831, Microlife AG®), respectively. After baseline measurements vital parameters were always collected 15 min before the next stimulation. For HR and RR the time points 0.75, 1.75, 2.75, 3.75, 5.75, 7.75, 9.75, 11.75, 23.75, 25.75, 27.75, 29.75, 31.75, 33.75, 35.75, 47.75, 49.75, 51.75, 53.75, 55.75, 57.75, 59.75, 71.75, 73.75, 75.75, 77.75, 79.75, 81.75, 83.75 and 95.75 h after drug administration were chosen. Blood pressure and Temp were measured 0.75, 1.75, 3.75, 7.75, 11.75, 23.75, 27.75, 31.75, 35.75, 47.75, 51.75, 55.75, 59.75, 71.75, 75.75, 79.75, 83.75 and 95.75 h after drug administration.

#### Behaviour score

Behaviour was scored using MBS [53]. Scores of − 10 to 14 were possible, with negative values indicating sedation and positive scores indicating excitation. Behaviour was scored at the same time points as HR, RR; SAP; MAP; DAP and Temp and always in exactly the same chronological order: RR, vocalization, posture, appearance from outside the play pen and interactive behaviour, HR, arterial blood pressure, restraint, Temp and response to noise from inside the play pen. All data were evaluated by the same investigator and needed approximately the same amount of time.

#### Gastrointestinal passage time

The GIPT was evaluated by feeding medical charcoal in a dosage of 0.5 mg kg^− 1^ with the food in the morning of trial day 1 and 3 and by measuring the time until black faeces were detected.

#### Methadone plasma levels and pharmacokinetic data

Venous blood samples were collected prior to drug administration for baseline and 1 min, 0.25, 0.5, 1, 2, 4, 6, 12, 24, 36, 48, 72, 73, 76, 80 and 96 h after drug administration. Blood samples (2.6 ml) were collected into lithium-heparinized tubes and were immediately centrifuged for 2 min at 10000 g. Plasma was separated and stored at − 80 °C until analysis.

Methadone plasma concentrations were measured with GC-MS with deuterated internal standard. Therefore, serum was mixed with deuterated, internal standard (Methadone-D_9_), was alkalised with sodium hydroxid and was extracted with 1-chlorbutane. After phase separation by centrifugation, the organic extract was transferred into a conic vial and evaporated with nitrogen to dryness. The residue was dissolved in ethyl acetat and transferred to autosampler vials for the GC/MS analysis. The analysis was performed with a gas chromatograph (Agilent Technologies 66,890 N), combined with an 5975C inert mass spectrometer and 7683B autosampler. The quantification was performed as followed:

The peak areas of the monitored ions were automatically calculated by the Agilent ChemStation-data system. Peak area ion ratios for the target-ion were used to calculate concentrations of methadone and qualifier ion were used to identify methadone and the internal standard (Methadone-D_9_). Five calibrators (in serum) run with each batch were used to generate the calibration curve, which was calculated using least-squares equitation.

The method was linear from 0,005 mg/l to 0,120 mg/l methadone (Limits of quantification).

Pharmacokinetic data were determined using a pharmacokinetic software (WinNonlin version 6.4; Pharsight Corp., MO, USA), including plasma levels from 0.25 to 96 h after drug administration, using Gauss-Newton Lavenberg and Hartley algorithm [54].

### Statistical analysis

Before starting the experiment, sample size was calculated via a priori power analysis using G-Power with an effect size of 1.15° Celsius and a power of 0.8.

Data of 7 dogs were analysed with SAS Enterprise Guide 7.1 and graphs were plotted with GraphPad Prism 6. Tests of normality were performed using Shapiro-Wilk-, Kolmogorov-Smirnov-, Cramer-von Mises and Anderson-Darling-tests as well as q-q plots. Parametric data (nociceptive threshold data, HR, RR, SAP, MAP, DAP and Temp) were compared between treatments and between time points using two-way ANOVA for repeated measurements and one-way ANOVA for repeated measurements, respectively. These data are presented as mean ± standard deviation (SD). MBS and GIPT were analysed by Wilcoxon singed rank test and are presented as median with minimum and maximum. Statistical significance was set at α = 5%.

## Data Availability

The datasets used and/or analysed during the current study are available from the corresponding author on reasonable request.

## Abbreviations

CRI: Constant rate infusion
M: Treatment group methadone
P: Treatment group placebo
%TE: Percentage thermal excursion
BL: Baseline
MT: Mechanical threshold
NMDA: N-methyl-d-aspartate
SD: Standard deviation
RR: Respiratory rate
HR: Heart rate
SAP: Systolic arterial blood pressure
DAP: Diastolic arterial blood pressure
MAP: Mean arterial blood pressure
Temp: Rectal temperature
MBS: Multimodal behaviour score
GIPT: Gastrointestinal passage time
°C: Degree Celsius
V_d_: Volume of distribution
Cl: Clearance
t_0.5_: Terminal half-life
LOQ: Limits of quantification
GC-MS: Gaschromatography-massspectroscopy
mmHg: Millimetre mercury
N: Newton
T_S_: Skin temperature
T_R_: Reaction temperature
T_C_: Cut-out temperature
